# Core outcome domain sets for clinical trials in epidermolysis bullosa — a COSEB protocol to achieve consensus on “what” to measure

**DOI:** 10.1186/s13063-025-09052-w

**Published:** 2025-10-09

**Authors:** Eva W. H. Korte, Peter C. van den Akker, Dimitra Kiritsi, Jan Kottner, Anna M. G. Pasmooij, Cecilia A. C. Prinsen, Verena Wally, Tobias Welponer, Phyllis I. Spuls, Martin Laimer, Maria C. Bolling

**Affiliations:** 1 Department of Dermatology, UMCG Center of Expertise for Blistering Diseases, University Medical Center Groningen, University of Groningen, Groningen, The Netherlands; 2 Department of Genetics, UMCG Center of Expertise for Blistering Diseases, University Medical Center Groningen, University of Groningen, Groningen, The Netherlands; 3 Department of Dermatology, Medical Center, Faculty of Medicine, University of Freiburg, Freiburg, Germany; 4 First Department of Dermatology, Faculty of Medicine, Aristotle University of Thessaloniki, Thessaloniki, Greece; 5 Charité-Universitätsmedizin Berlin, Institute of Clinical Nursing Science, Berlin, Germany; 6 Dutch Medicines Evaluation Board, Utrecht, The Netherlands; 7 Division of Pharmacoepidemiology and Clinical Pharmacology, Utrecht Institute for Pharmaceutical Sciences (UIPS), Utrecht University, Utrecht, The Netherlands; 8 CHORD COUSIN Collaboration, Amsterdam Public Health, Infection and Immunity, Department of Dermatology, Academic Medical Center, Amsterdam UMC, University of Amsterdam, Amsterdam, The Netherlands; 9 Department of Dermatology and Allergology and EB House Austria, University Hospital of the Paracelsus Medical University, Salzburg, Austria; 10 Department of Dermatology, Academic Medical Center, Amsterdam UMC, University of Amsterdam, Amsterdam, The Netherlands; 11 Amsterdam Public Health, Infection and Immunity, Academic Medical Center, University of Amsterdam, Amsterdam, The Netherlands

**Keywords:** Core outcome set, Epidermolysis bullosa, Clinical trial, Outcome measurement

## Abstract

**Background:**

Epidermolysis bullosa (EB) comprises a heterogeneous group of rare, genetic blistering diseases. The wide variety in EB trial outcomes limits the comparability of outcomes and, consequently, the implementation of the best available treatment options. A core outcome set (COS) is a minimum set of outcomes that should be measured in all clinical trials, comprising *what* should be measured (i.e., outcome domains) and *how* it should be measured (i.e., outcome measurement instruments). This enables standardization of outcome measurement aiming at improving the comparability and quality of research.

**Methods:**

The Core Outcome Set for Epidermolysis Bullosa (COSEB) initiative aims to develop COSs for use in clinical trials for the four major EB types: EB simplex, junctional EB, dystrophic EB, and Kindler EB. This protocol focuses on the development of core outcome domain sets — outlining *what* should be measured in EB clinical trials. Involved stakeholders are patients and patient representatives, clinicians, researchers, methodologists, industry representatives, regulators, health technology assessors, and payers. In the initial part, working groups are formed to define long lists of candidate outcome domains for the four major EB types. Potentially relevant outcome domains will be identified based on scoping literature reviews and qualitative studies. Following consultations with a stakeholder advisory panel, a short list of candidate outcome domains will be subject to voting in Delphi consensus procedures. Finally, the definitive core outcome domain sets for the four major EB types and, if indicated, any overarching core outcome domain sets, will be confirmed in consensus meetings. The project Has been prospectively registered in the COMET registry for COSs on 23 October 2017 (registration number 1033).

**Discussion:**

This protocol provides guidance to ensure a systematic, transparent, and comprehensible approach of COSEB. The final core outcome domain sets are supposed to serve as the minimum sets of *what* to measure in future EB trials. This will provide the basis for the subsequent outcome measurement instrument selection. Particularly in this rare disease with inherently small-sized study cohorts, this will facilitate the incorporation of meaningful outcomes and pooling of data, ultimately enhancing optimal treatment for EB.

**Trial registration:**

This study Has been prospectively registered in the COMET database on 23 October 2017 and updated on 24 January 2022 (registration number 1033 https://www.comet-initiative.org/studies/details/1033).

**Supplementary Information:**

The online version contains supplementary material available at 10.1186/s13063-025-09052-w.

## Background

Epidermolysis bullosa (EB) comprises a group of rare genetic disorders characterized by the formation of blisters and erosions on the skin and mucous membranes upon minimal trauma [1, 2]. EB Has an estimated incidence of 19.6–67.8 per million live births [3–6] and is classified into four major types: EB simplex (EBS), junctional EB (JEB), dystrophic EB (DEB), and Kindler EB (KEB) [1]. These types present with heterogeneous genetic and clinical hallmarks, ranging from palmoplantar blistering improving with age to development of generalized cutaneous and multisystemic manifestations, leading to severe morbidity and limited lifespan [1, 7, 8].

Over the years, increasingly important clinical research focuses are the developments of novel or repurposed treatment strategies for patients with EB [9–11]. However, EB clinical research is hitherto characterized by remarkable heterogeneity of outcomes and outcome measurement instruments [12–14]. Wide variety in *what* (i.e., outcome domains) and *how *(i.e., outcome measurement instruments) to measure hinders adequate cross-comparison of treatment effects [15]. Particularly, in the setting of a rare disease like EB, this has significant implications on determining the efficacy and safety of treatments, thus impeding progress in therapy development.

Recognizing the urgent need to address these challenges, the Core Outcome Set for Epidermolysis Bullosa (COSEB) initiative was founded to work towards developing core outcome sets (COSs) for EB clinical trials concerning the four major EB types [16]. A COS is a standardized set of the most important outcomes, agreed upon by key stakeholders, which should be assessed and reported as a minimum in future clinical trials involving a particular condition [15], alongside the outcomes that are of specific interest to the investigator. The use of COS is supported by the Consolidated Standards of Reporting Trials (CONSORT) and Standard Protocol Items: Recommendations for Interventional Trials (SPIRIT) guidelines [17, 18], as well as the Cochrane methodology of reviews of interventions [19]. Developing a COS for EB is essential to ensure that measurement is meaningful, comparable, and evidence based to guide clinical and regulatory decision-making and expedite clinical translation of novel therapies [15, 20].

### Aims and objectives

The aims of COSEB are to work towards uniform and optimum reporting and measurement of core outcomes in EB trials, collaboratively involving key stakeholders. Specifically, the objectives of COSEB are (first) to reach international multistakeholder consensus on the most important (i.e., “core”) outcome domains for each of the major EB types and (second) to reach international multistakeholder consensus on the outcome measurement instruments to measure the selected core outcome domains. A final COS comprises the combination of these two steps [21]. As therapy development in EB can target multiple EB types together, an overarching COS may additionally be proposed based on common core domains across EB types. The goal of the current protocol is to provide guidance for the determination of core outcome domain sets for each of the four EB types.

## Methods

### Scope

- *Condition*: Patients of any age and background diagnosed with EB.
- *Intervention*: Any local and systemic therapy for patients with EB. Procedural interventions (e.g., dressings, laser, microneedling, surgery) are not considered based on the differences in focus of outcome domains compared to pharmacological studies [12].
- *Context*: Interventional clinical trials of any phase, worldwide.

### Protocol methodology

COSEB is affiliated with the CHORD COUSIN Collaboration (C3) [22] and has been prospectively registered with Core Outcome Measures in Effectiveness Trials (COMET) [23]. The current protocol is based on the COMET Handbook [15] and was inspired by Harmonizing Outcome Measures for Eczema (HOME) road map [21], HOME implementation road map [24], outcome measures in rheumatology (OMERACT) uptake recommendations [25], and other C3-affiliated and dermatology-specific COS groups [26–32]. The protocol follows the Core Outcome Set-STAndardised Protocol Items (COS-STAP) [33].

### Terminology and definitions

Currently, various terms and concepts are used in the field of COS development. Terms with corresponding definitions used in this protocol are depicted in Table 1. The level of detail (i.e., granularity) in describing outcome domains significantly influences the effectiveness and utility of COSs [38]. More granular domains (e.g., “erythema” compared to “cutaneous inflammation”) can lead to a higher number of candidate domains in the consensus process but have the benefit of specific and focused outcome measurement instruments, while less granular domains may risk varied interpretations but simplify consensus. COSEB aims for optimal granularity to better facilitate outcome measurement instrument selection, achieved through working group discussions and COS expert consultations [36]. Additionally, ensuring no conceptual overlap between domains, such as “wound closure” within the broader “wound healing,” is a priority.

**Table 1:** COSEB protocol terms and definitions

Term	Definition	Examples
Outcome domain	In the context of a clinical trial, it refers to *what* is being measured on trial participants to examine the effect of exposure to a health intervention [34].	Wound healing, pruritus, EB-related quality of life.
Candidate core outcome domain	An outcome domain that will be considered for inclusion in the core outcome domain set with a corresponding definition.	*-*
Core outcome domain	An outcome domain as part of the core outcome set.	*-*
Core outcome domain set	A set of outcome domains constituting the minimum that should be measured in a specified research field — the “what” to measure.	A core domain set for hidradenitis suppurativa trial outcomes (HiSTORIC) [28].
Outcome measurement instrument	An outcome measurement instrument refers to *how *the outcome domain is being measured. It is a tool to measure the outcome of interest. The instrument can be a single question, a questionnaire, a score obtained through physical examination or image, a laboratory measurement, etc [34].	Numerical rating scale (NRS) or the Investigator Global Assessment (IGA) score.
Core outcome set	Agreed standardized set of outcomes comprising the core outcome domains (*what* to measure) and corresponding core outcome measurement instrument (*how* to measure), which should be measured and reported, as a minimum, in all clinical trials in a specific disease or trial population [21].	The core outcome set of Harmonizing Outcome Measures for Eczema (HOME) [35].
Fully specified outcome	The entirety of domain, outcome measurement instrument, metric, aggregation, and time frame to be investigated in a study or to be used in a systematic review [36, 37].	Proportion of subjects with complete closure (complete reepithelialization without drainage) of a target wound within 45 days of treatment compared to control based on clinical assessment by the investigator.

### Stakeholders

To ensure outputs are deemed relevant by all stakeholders in EB therapy development, COSEB aims to include individuals with various professional, demographic, and geographical backgrounds. The following stakeholder groups are involved:

- *Patients and patient representatives*: Aiming to conduct patient-centered research, patients and patient representatives are regarded as key participants in COSEB. Parents of pediatric patients, patients aged 16 years and over, and patient advocates are approached and informed in person or via email through EB expertise centers and through the EB-specific patient organizations (DEBRAs).
- *EB experts*: This group involves researchers, clinicians (i.e., physicians such as dermatologists, pediatricians, and nurses), and investigators involved in EB research or care. These experts are identified through the EB Clinet expert database [39], previous collaborations, author lists of relevant publications, and at congresses.
- *Methodologists*: The specific methodologist profile for COSEB concerns people experienced in COS research, EB trial design and outcome measurement instrument development, and whether EB specific or in general. These stakeholders are identified through collaboration with C3, author lists of relevant publications, and at congresses.
- *Industry*: Representatives of companies with an interest and/or experience in developing or evaluating drugs for EB are identified through previous collaboration and online searches.
- *Regulators*: Representatives of regulatory bodies involved in regulatory requirements of EB trials and marketing authorization of medicines for EB are identified through contacts at regulatory agencies (e.g., EMA, FDA, PMDA) and through previous scientific advice meetings.
- *Health technology assessors and payers*: Representatives of health technology assessors and payers, involved in health technology assessment and reimbursement of medicines for EB, are identified through contacts with previous collaboration and online searches.

### COSEB Consortium

There are three groups distinguished within the COSEB Consortium, which operate autonomously from each other:

- *The steering committee* (SC), comprised of its founders (E. K., P. A., D. K., A. P., V. W., T. W., M. L., M. B.), oversees project scope, coordination, and alignment of the working groups and advisory panel. The current composition of the SC consists of clinicians, researchers, and regulators. The SC is responsible for familiarizing the consortium with the COS methodology by publishing protocols and organizing strategic meetings and workshops. It oversees protocol adherence and data organization and provides document templates. Moreover, the SC is responsible for preparing and organizing the logistical part of the Delphi procedures and consensus meetings. Lastly, it ensures final publication and dissemination of project outputs.
- *The working groups* (WGs) are formed for all four major types of EB (EBS, DEB, JEB, and KEB) and are composed of mixed stakeholders responsible for compiling “long lists” of outcome domains. Each WG is subsequently requested to create a “short list” through refinement of collected outcome domains, addressing the comments provided by the advisory panel. The WG will be responsible for the content of the candidate outcome domain lists and analyzing the Delphi and consensus meeting outputs. Each WG includes a chair, a vice-chair, and members from different stakeholder groups with EB experience. WG members are not allowed to participate as voters in the Delphi procedure focusing on their respective EB type, given their responsibility for preparing the short list and analyzing the Delphi data. However, they do have a final vote during the consensus meeting. If a WG remains unfilled, its task may be postponed, merged with another WG, or modified per the chair’s and SC’s decision.
- *The advisory panel* (AP) is composed of mixed stakeholders representing the six different stakeholder groups important to the COSEB initiative. The AP reviews the long list of outcome domains and their definitions compiled by the WGs and provides structured feedback on the relevance based on a predefined checklist composed by the steering committee.

COSEB was initiated with a kick-off meeting on April 12, 2023, to engage stakeholders globally [16]. A total of 104 participants from 24 countries attended this meeting, representing multiple continents and stakeholder roles. A post-meeting survey reflected strong support for the COS approach and willingness to collaborate. Guided by the SC, consortium members subsequently organized themselves into WGs for EBS and DEB and the AP. Following this, the first COSEB workshop was organized on December 15, 2023, with 50 consortium members to familiarize themselves with established COS methodology, connect, and start up the WGs’ activities. The COSEB road map was further refined, and invitations for additional COSEB Consortium members were extended to the EB community [40].

### Conflict of interest

All consortium members are requested to disclose competing interests through filling out a conflict of interest (COI) disclosure form before actively participating in COSEB (*Additional file 1*). COIs are reviewed by the SC and will be updated when new potential COIs arise. Experts with relevant COIs must constitute a minority within WGs. All conflicts will be transparently disclosed and their potential impact on decision-making processes throughout the consensus development considered, with all COIs of participating stakeholders published in the final consensus statement for full transparency.

### Patient and public contribution

Patient and public representatives are involved throughout the study [41]. Additionally, tailored information and training by pre-meetings and plain language summaries will be proactively provided following the People and Patient Participation, Involvement and Engagement (PoPPIE) recommendations [42].

## Design

**Fig. 1:**
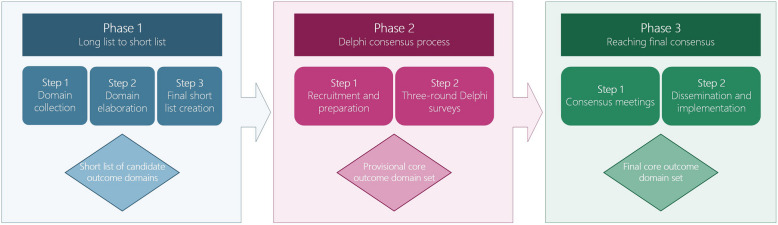
COSEB’s stepwise consensus process for developing core outcome domain sets for clinical trials. Legends: Figure depicts a flowchart of the three phases for developing core outcome domain sets for clinical trials focusing on the four major epidermolysis bullosa (EB) types, including EB simplex, junctional EB, dystrophic EB, and Kindler EB

The COSEB protocol consists of a multiphase approach (Fig. 1). Phase 1 consists of several steps to create short lists of candidate outcome domains, Phase 2 comprises Delphi consensus processes to achieve preliminary consensus, and Phase 3 aims to achieve final consensus on the core outcome domain sets for the different EB types through consensus meetings and dissemination and implementation strategies.

### Phase 1: Long list to short list

#### Step 1: Domain collection

##### Reviews

A scoping review of outcome measurements in EB clinical research from January 1991 to September 2022 has been published by the SC [12]. Out of 207 studies, 1280 outcomes were identified and assigned to 80 overarching outcome domains (*Additional file 2*). An additional systematic literature search in MEDLINE (PubMed) will be conducted by the WGs to update the initial scoping review search and to include English studies reporting patient perspectives on outcomes (e.g., surveys and qualitative studies). Identified treatment needs will be stratified per EB type. The WGs will review these previously reported outcome domains and may reallocate and/or combine them iteratively to broader outcome domains relevant to the specific EB type.

##### Patient interviews

To ensure consideration of patient perspectives, WGs will organize interviews involving patients with EB of any subtype with a minimum age of 16 years and caregivers. After ethics approval, recruitment will involve collaboration with DEBRA patient organizations and EB expertise centers. Participants will be purposively sampled to ensure diversity in age, sex, education, geography, clinical trial participation, and EB types until saturation. Informed consent will be obtained from all participants. The interviews will employ a semi-structured format with open-ended questions, complemented by a think-aloud component where patients review and comment on the predefined outcome domain list and definitions developed by the WGs [43]. Interview guides will be developed [44], and interviewing researchers will undergo training. Sessions, either in person or virtually, will be audio recorded. Content analysis, supported by ATLAS.ti software, will be applied to identify and extract potential outcome domains. These domains will then be assigned to established domains or formulated into new domains, as determined by the WGs.

#### Step 2: Domain elaboration

##### Domain selection

 All collected outcome domains from the previous step will be checked by the WGs, and duplicates will be removed. The WGs will classify and prioritize the outcome domains based on outputs of the literature reviews and interviews of the previous step. The subsequent long list of candidate outcome domains will be supplemented with corresponding definitions based on the reviews and outputs of the interviews. These definitions should contain a medical/scientific definition as well as a plain language definition focused on patients [45].

##### Advisory panel consultations

The long list of outcome domains and their definitions will be sent to the AP for review. The AP will verify that the domains on the long list are relevant to scope, comprehensible, clear, and feasible (i.e., measurable) [46]. Additionally, the methodologists in the AP will check if the domains have the same level of granularity and are conceptually distinct. The AP may propose adjustments regarding wording and definitions and/or propose additional outcome domains. Lastly, the SC will review if the formal requirements per protocol are met. If needed, debatable outcome domains in light of these requirements are discussed at WG and SC meetings, with WGs having final decision authority.

#### Step 3: Final short list creation

WGs will refine the long list into a short list of candidate outcome domains, providing arguments for their selection. COSEB aims for the short list to contain a maximum of approximately 30 domains. This target is based on previous experience of other COS initiatives [47]. Moreover, limiting the maximum number of domains is expected to enhance the focus on relevant and critical domains and reduce the Delphi voting burden [48]. Exceptions exceeding this maximum number require justification by the WG. The final short list serves as a basis for the Delphi consensus process.

### Phase 2: Delphi consensus process

The Delphi method is a structured and systematic process that is used to systematically gather stakeholder opinions and achieve consensus through multiple rounds of questionnaires, with controlled feedback provided between each round [49]. It enables anonymous and controlled deliberations, allowing participants to refine their opinions while being influenced by the collective insights of the group [49]. The Delphi study will be carried out online using specialized software (REDCap) and reported following guidance on conducting and reporting Delphi studies [50].

#### Step 1: Recruitment and preparation

The Delphi panel consists of all stakeholders with experience in EB and/or COS development. WG members are not allowed to participate as voters in their EB-type specific Delphi survey but are permitted to all other Delphi surveys on complementary EB types. All eligibility criteria per stakeholder group are provided in Table 2. Though no specific guideline exists, a minimum of 100 Delphi participants is targeted, with at least 20 participants per stakeholder group [51]. Recruitment will be done through consortium contacts, patient organization networks, author lists of relevant publications, expert databases [39], online searches, at congresses, and through social media outreach. Further invitations are expected to be extended by the “snowballing” technique.

**Table 2:** Eligibility criteria for Delphi consensus process

Stakeholder role	Inclusion criteria
All participants	Agreed to participate in all Delphi rounds, provided informed consent
Patient(s) (representatives)	A patient diagnosed with any type of EB, minimum age of 16 years, caregiver of a patient, and/or a patient advocate
Experts	A minimum of 3 years of experience treating patients with EB and/or at least one publication in the field of EB
Industry	A minimum of 3 years of experience in the field of EB therapy development and involvement in at least one clinical trial
Regulators	Minimum of 3 years of experience in assessment and/or approval in EB clinical trial and/or medicinal products
Health technology assessors and payers	Minimum of 3 years of experience in health technology assessment and/or assessment for reimbursement

The SC will organize the recruitment and preparation for the Delphi rounds. Participating stakeholders will receive targeted background information about the scope, rationale, and the required steps at least 2 weeks prior to the start of the Delphi rounds. Additionally, pre-meetings will be organized by the SC for participants to explain the process in detail and in lay language for patients. Demographic information, including the panelists’ stakeholder role, amount of experience with EB, country of residence, and relevant conflicts of interest, will be recorded. The EB subtype of participating patients will also be recorded. All personalized data will be available to the WG members analyzing the Delphi outputs, but data that is shared with panelists or published will be aggregated.

#### Step 2: Three-round Delphi surveys

Three Delphi rounds are planned. In the first round, votes and arguments of Delphi panelists regarding the importance of proposed outcome domains will be collected, and missing domains that are being considered critically important for COS can be proposed. Round 1 group responses of outcomes on which no consensus has been obtained, including arguments provided by panelists, will be presented to panelists for re-voting in the second round, supplemented by the newly proposed outcome domains [52]. The third round confirms the voting choices, providing personal and group responses from the previous rounds and a final chance for adjustments. Results per round will be communicated to stakeholders through both numerical summaries and visual representations (e.g., graphically in percentages). The outcome domains will be presented randomly each round to avoid any influence of display order on the evaluation of the domains [53]. If deemed suitable, the domains listed in the Delphi rounds may be combined or subdivided to achieve appropriate granularity.

There is no reference standard about the appropriate level of consensus for COS Delphi studies [54–56]. The cut-off percentage for this protocol is set at 80%, indicating a strong consensus. Consensus definitions are provided in Table 3. If deemed necessary, alterations of these cut-off percentages are permitted provided that any changes are reported transparently with arguments during the Delphi procedure, consensus meeting, and in the final publication.

**Table 3:** Consensus definition

**Consensus “in”**	≥ 80% of all Delphi panelists score a 4 or 5, and ≤ 15% score a 1 or 2
Consensus “out”	≥ 80% of all Delphi panelists score a 1 or 2, and ≤ 15% score a 4 or 5
Undecided	Outcome domains not falling into either category among Delphi panelists may require additional Delphi rounds to achieve consensus and will be discussed in the consensus meeting

A 5-point rating scale will be used to rate the importance of each domain, where higher scores indicate higher levels of importance. To enhance accuracy, verbal labels to anchor all categories next to numeric labels in the rating scale will be used (1: “not at all” important, 2: “low” importance, 3: “moderate” importance, 4: “high” importance, and 5: “critical” importance) [56]. To limit missing data, the option “not able to answer this question” is available. Panelists are encouraged to provide arguments for their ratings in written form on each outcome domain.

Panelists will have 3 weeks to complete each Delphi round. Reminders will be sent weekly and extended to 4 weeks if response rates are below 70%. Response rates of each round will be documented. Only panelists who completed the previous round move on to the next, and priority will be given to those completing all three rounds for invitation to the final consensus meeting. However, additional participants may be invited to ensure comprehensive representation of expertise. This approach to consensus meeting participation will be clarified before the start of each Delphi round.

### Phase 3. Reaching final consensus

#### Step 1: Consensus meetings

All participants will be invited to a digital pre-meeting session explaining the goals and scope of the consensus meeting. This meeting will be held in a digital or hybrid format, led by independent and experienced moderators, and adjacent to international EB-related congresses (e.g., EB Clinet meetings, EB World Congress) to reach as many participants as possible. Results of the Delphi consensus process will be presented. Following this, participants will be divided into breakout room groups with similar stakeholder representation to discuss the prioritization of the remaining “undecided” outcome domains (Table 3). Subsequently, in an open plenary discussion, participants will be able to speak about their perspectives concerning the remaining “undecided” outcome domains. The decision on consensus of all outcome domains will be based on a vote, “in,” “out,” or “refrain from voting,” and is made when < 20% disagrees to include the outcome domain in the core outcome domain set, provided that no more than 80% of participants refrain from voting.

COSEB strives for no more than five core outcome domains in the core domain set per EB type, with an absolute maximum of seven core outcome domains. Setting a maximum number of core outcome domains is expected to enhance the feasibility of measuring and reporting the domains, thereby increasing the chances of uptake of the core outcome domains in future EB clinical trials. To achieve this target, consensus meeting participants will be allowed to adjust granularity where suitable by combining outcome domains. A final vote during the consensus meeting decides if the total set of included core outcome domains is accepted. If ≤ 20% disagree, this set will form the consensus. If > 20% disagree, a live survey regarding reasons of disagreement is distributed among the participants (too many domains, too few, other reason). If any of the reasons are supported by > 20% participants, discussions to either drop or add domains will be held, followed by re-voting seeking ≥ 80% agreement. The results of the Delphi study and final consensus meeting will be reported according to the Core Outcome Set-STAndards for Reporting (COS-STAR) checklist [54].

#### Step 2: Dissemination and implementation

The final COS will be disseminated by the SC to be used as widely and efficiently as possible. COSEB strives to publish all papers in open-access journals and present outputs whenever possible to peers in meetings. Relevant societies and networks in the field of EB, such as EB Clinet, European Joint Programme on Rare Diseases (EJP RD), European Reference Network-SKIN (ERN-SKIN) and DEBRA International, will be contacted to assist in disseminating COSEB’s outputs to the community.

This protocol follows uptake recommendations from HOME [24] and OMERACT [25]. COSEB involves various stakeholders, including patients, from the start to promote sustained global engagement. It strives to ensure user-friendly, universally applicable outcome domain sets, including plain language definitions alongside scientific ones. Moreover, recommendations on practical usage will feature in final consensus reports. Five years after consensus, the SC will evaluate the core outcome domain set impact on trial design by evaluating ongoing clinical trials on the uptake in trial endpoints. Investigators will be surveyed to gauge their experience with and attitude towards using the recommendations of COSEB.

### Future phases of COSEB

The scope of this protocol is limited to the development of the core outcome domain sets. Once these have been developed, the next step is reaching consensus on measurement instruments used to measure the core outcome domains, for which a separate protocol will be developed [15]. Existing outcome measurement instruments for each of the core domains will be subject to rigorous review following the COnsensus-based Standards for the selection of health Measurement INstruments (COSMIN) criteria [57, 58]. The outcome measurement instruments selected should demonstrate good performance for these COSMIN criteria such as content validity, internal structure (i.e., structural validity, internal consistency, and cross-cultural validity/measurement invariance), and other measurement properties (e.g., reliability, measurement error, criterion validity, hypotheses testing for construct validity, and responsiveness). New or adapted instruments may need to be developed if there is inadequate evidence supporting the selection of the available instruments [15]. Additionally, developing a COS for EB will establish an international network of key stakeholders with experience of contributing to a collaborative consensus study. This infrastructure could be leveraged to other settings such as selecting research priorities [59], standardized registry topics, and baseline characteristics [60, 61]. For example, in addition to developing COSs for the different EB types, COSEB intends to establish a minimum reporting set (i.e., the study and population (baseline) characteristics that should, as a minimum, be reported in every study) to further enhance comparability across clinical trials [62–65].

## Conclusions

The current protocol serves as a road map for the necessary steps to work towards more standardization and harmonization of outcome measurement in EB clinical trials. This approach holds the potential to increase the outcome comparability of future interventions for EB, thereby expediting therapy development and eventually improving treatment of EB worldwide.

## Supplementary Information

Additional file 1. COSEB COI form

Additional file 2. Outcome domain areas and outcome domains identified in the scoping review

## Data Availability

The data underlying this article will be shared on reasonable request to the corresponding author. The final core outcome set will be submitted for peer review and reported according to the COS-STAR.

## Abbreviations

AP: Advisory panel
C3: CHORD COUSIN Collaboration
COI: Conflict of interest
COMET: Core Outcome Measures in Effectiveness Trials
CONSORT: Consolidated Standards of Reporting Trials
COS: Core outcome set
COSEB: Core Outcome Set for Epidermolysis Bullosa
COSMIN: COnsensus-based Standards for the selection of health Measurement INstruments
COS-STAP: Core Outcome Set-STAndardised Protocol Items
COS-STAR: Core Outcome Set-STAndards for Reporting
DEB: Dystrophic epidermolysis bullosa
DEBRA: EB patient organization
DP: Delphi panel
EB: Epidermolysis bullosa
EBS: Epidermolysis bullosa simplex
EJP-RD: European Joint Programme on Rare Diseases
ERN-SKIN: European Reference Network-SKIN
HiSTORIC: Hidradenitis Suppurativa Core Outcomes Set International Collaboration
HOME: Harmonizing Outcome Measures for Eczema
JEB: Junctional epidermolysis bullosa
KEB: Kindler epidermolysis bullosa
OMERACT: Outcome measures in rheumatology
PoPPie: People and patient participation, involvement and engagement
SC: Steering committee
SPIRIT: Standard Protocol Items: Recommendations for Interventional Trials
WG: Working group
