# Supplementary material for: Core outcome domain sets for clinical trials in epidermolysis bullosa — a COSEB protocol to achieve consensus on “what” to measure
Source: Trials. 2025 Oct 9;26:399. doi: 10.1186/s13063-025-09052-w (PMC12512268; doi:10.1186/s13063-025-09052-w)
Supplement: Supplementary file 1 — Additional file 1. COSEB COI form [file 13063_2025_9052_MOESM1_ESM.docx]

# **Supplementary material**

**Additional file 1.** COSEB COI form

**COSEB DISCLOSURE FORM**

| **Date:** |  |
| --- | --- |
| **Name:** |  |
| **Stakeholder role:** |  |

In the interest of transparency, we ask you to disclose all potential competing interests listed below that are related to your participation in COSEB. Disclosure represents a commitment to transparency and does not necessarily indicate a bias. If you are in doubt about whether to list an interest, it is preferable that you do so.

Please declare the **nature** of the declared interest, the **timeframe** during which such interest occurred, as well as the **type** of activity

| Please specify the following potential interest regarding inclusion of outcome domains or outcome measurement instruments in the final core outcome set for any of the EB types: | | |
| --- | --- | --- |
| 1 | **Direct interests** |  |
| 1.a | **Employment**  Employment with industry focusing on drug development in any outcome domain relevant for EB) (i.e., a pharmaceutical company, a medical device company or the biotechnology sector)  (e.g., occupation, part-time or full-time, paid, or unpaid) | \| ☐ \| **None** \| \| --- \| --- \|  \|  \| \| --- \| \|  \| \|  \| |
| 1.b | **Consultancy or advisory role**  Consulting or advising industry focusing on drug development in any outcome domain relevant for EB.  (e.g., providing advice or participating in a(n) (scientific) advisory board, steering committee, or executive committee) | \| ☐ \| **None** \| \| --- \| --- \|  \|  \| \| --- \| \|  \| \|  \| |
| 1.c | **Financial interests**  Any other economic stake regarding an outcome domain or instrument related to (any of) the following:  - Holding of stocks and shares, stock options, stock warrants, equities, bonds, ownership, or partnership interest.  - Compensation, fees, honoraria, salaries, grants, or other funding (including rents, sponsorships, and fellowships) paid by Industry  - Intellectual property rights including patents, trademarks, know-how and/or copyrights relating to a medicinal product | \| ☐ \| **None** \| \| --- \| --- \|  \|  \| \| --- \| \|  \| \|  \| |
| 2 | **Indirect interests** |  |
| 2.a. | **Scientific interests**  **Interests regarding the scope of academic career and scientific projects**  (e.g., if your scientific work focuses on one particular outcome domain, or if you have been involved in the development of outcome measurement instruments in the field) | \| ☐ \| **None** \| \| --- \| --- \|  \|  \| \| --- \| \|  \| \|  \| |
| 3 | **Other indirect interests**  (e.g., any other conflicts you deem important to list as interest) | \| ☐ \| **None** \| \| --- \| --- \|  \|  \| \| --- \| \|  \| \|  \| |
| Please place an “X” next to the following statement to indicate your agreement: | | |
| ☐ | I certify that I have answered every question and have not altered the wording of any of the questions on this form. | |
