# Supplementary material for: Core outcome domain sets for clinical trials in epidermolysis bullosa — a COSEB protocol to achieve consensus on “what” to measure
Source: Trials. 2025 Oct 9;26:399. doi: 10.1186/s13063-025-09052-w (PMC12512268; doi:10.1186/s13063-025-09052-w)
Supplement: Supplementary file 2 — Additional file 2. Outcome domain areas and outcome domains identified in the scoping review [file 13063_2025_9052_MOESM2_ESM.docx]

**Additional file 2.** Outcome domain areas and outcome domains identified in the scoping review^12^

| **Outcome area** | **Outcome domain** |
| --- | --- |
| **Cutaneous manifestations** | Wound healing |
|  | Blister formation |
|  | Lesion characteristics and appearance |
|  | Total skin involvement |
|  | Skin resistance |
|  | Wound formation |
|  | Cancer formation |
|  | Infection |
|  | Blister healing |
|  | Lesion healing |
|  | Lesion formation |
|  | Healing of donor graft site |
|  | Scarring |
| **Safety** | Any adverse events |
|  | Toxicity |
|  | Infection as adverse event |
|  | Tolerability, tolerance |
|  | Antibody formation |
|  | Pain associated with treatment |
|  | Blister formation due to treatment |
| **Symptoms** | Pain associated with EB |
|  | Pruritus |
|  | Pain associated with activity |
|  | General symptoms |
| **Biochemical markers** | Protein expression in the skin |
|  | Systemic biochemical marker |
|  | Anchoring fibril presence |
|  | Epidermal and dermal characteristics |
|  | Gene expression |
|  | Chimerism |
|  | Brain connectivity |
| **Physical functioning** | Hand functioning |
|  | Ability to chew and swallow |
|  | Contractures |
|  | Daily functionality and disability |
|  | Intake and appetite |
|  | Ability to move |
|  | Plantar characteristics |
|  | Energy and activity levels |
| **Quality of life** | EB-specific quality of life |
|  | General quality of life |
|  | Dermatological quality of life |
|  | Quality of life associated with symptoms |
| **Clinical assessment** | Overall disease severity |
|  | Global assessment by investigator |
| **Resource use** | Required surgical interventions |
|  | Pain medication usage |
|  | Wound care usage |
|  | Duration of hospital admissions |
|  | Other medication usage |
|  | Duration of wound care |
|  | Costs |
|  | Duration of surgical intervention |
|  | Hospitalization |
| **Treatment characteristics** | Graft take |
|  | Successful implant and bone integration |
|  | Feasibility of wound treatment |
|  | Risk due to treatment |
|  | Successful esophageal dilatation |
|  | Successful gastrostomy placement |
|  | Willingness to continue treatment |
| **Patient satisfaction** | Patient reported satisfaction and benefit |
|  | Comfort |
| **Development status** | Weight and height status |
|  | Nutritional status |
|  | Pubertal status |
| **Extracutaneous manifestations** | Oral and dental health |
|  | Digestion and defecation |
|  | Esophageal strictures |
|  | Corneal symptoms |
| **Psychosocial functioning** | Mental health |
|  | Sleep |
|  | Family life |
|  | Psychological impact |
|  | Well-being |
|  | Social functioning |
|  | Support |
| **Death/survival** | Overall survival |
|  | Event-free survival |
|  | Treatment related mortality |
